# Exploring the evolution of circular polarized light backscattered from turbid tissue-like disperse medium utilizing generalized Monte Carlo modeling approach with a combined use of Jones and Stokes–Mueller formalisms

**DOI:** 10.1117/1.JBO.29.5.052913

**Published:** 2023-11-21

**Authors:** Ivan Lopushenko, Oleksii Sieryi, Alexander Bykov, Igor Meglinski

**Affiliations:** a University of Oulu, Opto-Electronics and Measurement Techniques Unit, Faculty of Information Technology and Electrical Engineering, Oulu, Finland; b Aston University, College of Engineering and Physical Sciences, Birmingham, United Kingdom

**Keywords:** circularly polarized light, Monte Carlo, Stokes vector, Jones–Mueller approach, polarimetry, turbid tissue-like scattering medium

## Abstract

**Significance:**

Phase retardation of circularly polarized light (CPL), backscattered by biological tissue, is used extensively for quantitative evaluation of cervical intraepithelial neoplasia, presence of senile Alzheimer’s plaques, and characterization of biotissues with optical anisotropy. The Stokes polarimetry and Mueller matrix approaches demonstrate high potential in definitive non-invasive cancer diagnosis and tissue characterization. The ultimate understanding of CPL interaction with tissues is essential for advancing medical diagnostics, optical imaging, therapeutic applications, and the development of optical instruments and devices.

**Aim:**

We investigate propagation of CPL within turbid tissue-like scattering medium utilizing a combination of Jones and Stokes–Mueller formalisms in a Monte Carlo (MC) modeling approach. We explore the fundamentals of CPL memory effect and depolarization formation.

**Approach:**

The generalized MC computational approach developed for polarization tracking within turbid tissue-like scattering medium is based on the iterative solution of the Bethe–Salpeter equation. The approach handles helicity response of CPL scattered in turbid medium and provides explicit expressions for assessment of its polarization state.

**Results:**

Evolution of CPL backscattered by tissue-like medium at different conditions of observation in terms of source–detector configuration is assessed quantitatively. The depolarization of light is presented in terms of the coherence matrix and Stokes–Mueller formalism. The obtained results reveal the origins of the helicity flip of CPL depending on the source–detector configuration and the properties of the medium and are in a good agreement with the experiment.

**Conclusions:**

By integrating Jones and Stokes–Mueller formalisms, the combined MC approach allows for a more complete representation of polarization effects in complex optical systems. The developed model is suitable to imitate propagation of the light beams of different shape and profile, including Gaussian, Bessel, Hermite–Gaussian, and Laguerre–Gaussian beams, within tissue-like medium. Diverse configuration of the experimental conditions, coherent properties of light, and peculiarities of polarization can be also taken into account.

## 1 Introduction

Recent advances of the biomedical polarimetry have clearly demonstrated that circularly polarized light (CPL) can be effectively used for overall characterization of biological tissues with optical anisotropy,^1^^–^^3^ including detection of the senile Alzheimer’s plaques^4^^,^^5^ and quantitative evaluation of the cervical intraepithelial neoplasia.^6^^,^^7^ Proper exploration of the CPL-tissue interaction requires accurate self-consistent descriptive simulation tools.^1^^,^^8^^,^^9^ Monte Carlo (MC) based approaches are widely recognized as efficient tools for analyzing light scattering by biological tissues and turbid medium.^10^^–^^14^ In biophotonics, MC methods, such as MCML,^15^ created by L. Wang and S. Jacques, were originally designed to simulate scalar light transport within turbid scattering medium^16^^,^^17^ and were fundamentally relying on the radiative transfer equation (RTE).^18^^–^^20^ As significant role of polarized light in extending diagnostic capabilities of biomedical tools became apparent,^21^^,^^22^ MC methods evolved accordingly resulting in many practical and popular tools particularly developed by Ramella-Roman, Prahl, and Jacques,^23^^,^^24^ Hielscher,^25^^,^^26^ Wang,^27^ and Xu.^28^ Fundamental ground for these polarized MC approaches was established by the vector radiative transfer equation (VRTE), which represents a system of equations for each Stokes parameter and can be rigorously derived from the Maxwell electromagnetic theory.^29^^–^^31^ At the same time, an approach based on the iterative solution to Bethe–Salpeter (BS) equation ^19^^,^^32^^–^^34^ utilizing Jones vector formalism has been demonstrated to be effective for polarization tracking of MC-photons within turbid tissue-like medium and simulation of coherent backscattering.^13^^,^^14^^,^^35^^–^^39^ Recently, it has been shown on the fundamental level that VRTE- and BS-based approaches are equivalent under certain conditions.^40^ Advantages of the BS-based approach involve a direct relation to the analytic Milne solution and intuitive physical interpretation of the multiple scattering process via ladder diagrams.

**Fig. 1:**
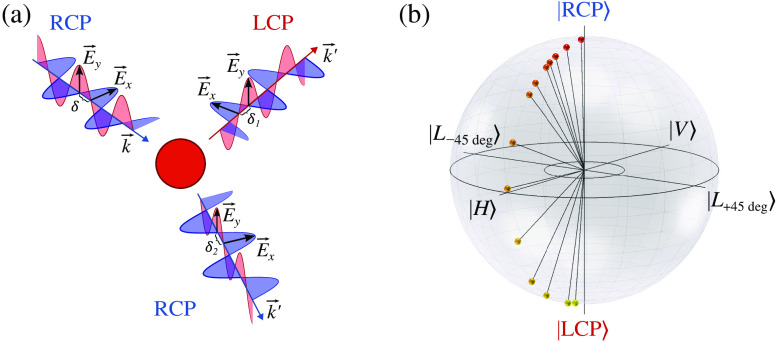
(a) Physics of the helicity flip: when RCP light is scattered in forward direction its helicity is preserved, whereas for backscattered light its polarization state is changed to LCP. (b) Degenerate polarization states $\left|H\rangle ,|V\rangle ,|L_{+45\;\deg}\rangle ,|L_{-45\;\deg}\rangle ,|\mathrm{RCP}\rangle ,|\mathrm{LCP}\right\rangle$ (defined in Sec. 2.1) and helicity flip (polarization state crossing the equator) depicted on the Poincaré sphere.

Modern implementations of the polarization-resolved MC^14^^,^^41^ aim to provide a comprehensive description of polarized light scattering with either Jones or Mueller formalism, depending on the representation of the polarization state.^42^ Most interest is shown in CPL which, unlike linearly polarized light, possesses a unique sense of directional awareness allowing to determine if light was forward or backscattered due to its intrinsic angular momentum associated with helicity^35^^,^^39^^,^^43^ [see Fig. 1(a)]. This peculiar property of CPL is a manifestation of anisotropy of scattering^8^ and is also known as polarization memory.^44^^–^^46^ Stokes vector polarimetry approach with the Poincaré sphere as a graphical tool is viewed as one of the most fitting instruments for light characterization with account for helicity [see Fig. 1(b)].

In this work, we address the conservation of the polarization memory and penetration depth of the CPL scattered in turbid tissue-like medium. We introduce an MC modeling approach specially developed to unify and generalize BS-based simulation of linearly, circularly, and/or elliptically polarized light propagation. For the first time we express the BS-based MC model in terms of the Stokes–Mueller formalism and show that our approach efficiently allows to compute Jones and Stokes vectors, Mueller matrix components, and all degrees of polarization. We explore the evolution of the CPL depolarization while propagating within turbid tissue-like scattering medium and consider the dynamic binding of circular polarization memory with the helicity flips occuring along the light path length within the medium.

## 2 Theory

### 2.1 Relation Between Stokes and Jones Formalism

Stokes vector is traditionally defined for the fully polarized light in the following form:^43^

$$(\begin{array}{c} I_{p}\\ Q_{p}\\ U_{p}\\ V_{p} \end{array})=\frac{1}{2}(\begin{array}{c} E_{x}E_{x}^{*}+E_{y}E_{y}^{*}\\ E_{x}E_{x}^{*}-E_{y}E_{y}^{*}\\ E_{x}E_{y}^{*}+E_{y}E_{x}^{*}\\ j(E_{x}E_{y}^{*}-E_{y}E_{x}^{*}) \end{array}).$$ (1)

Here, j denotes the imaginary unit, asterisk corresponds to complex conjugation, $E_{x}={\tilde{E}}_{0x}e^{j\delta_{x}}e^{j\omega t}$, $E_{y}={\tilde{E}}_{0y}e^{j\delta_{y}}e^{j\omega t}$ is a complex electric field of the plane wave propagating along z axis (wave vector $k↑↑e_{z}$), with ${\tilde{E}}_{0x},{\tilde{E}}_{0y}$ being wave real amplitudes multiplied by complex $e^{-j\mathrm{kr}}$ factor with position r, $\delta_{x},\delta_{y}$ corresponding to phases, and $E_{0x}={\overset{˜}{E}}_{0x}e^{j\delta_{x}}$, $E_{0y}={\overset{˜}{E}}_{0y}e^{j\delta_{y}}$ being wave complex amplitudes. Both complex fields $E_{x}$, $E_{y}$ can be decomposed into real (R) and imaginary (I) parts:

$$(\begin{array}{c} E_{xx}\\ E_{xy} \end{array})=R(\begin{array}{c} E_{0x}\\ E_{0y} \end{array}),\;(\begin{array}{c} E_{yx}\\ E_{yy} \end{array})=I(\begin{array}{c} E_{0x}\\ E_{0y} \end{array}).$$ (2)

In terms of Jones formalism, it can be written as

$$|J\rangle =(\begin{array}{c} E_{0x}\\ E_{0y} \end{array})=(\begin{array}{c} E_{xx}\\ E_{yx} \end{array})+j(\begin{array}{c} E_{yx}\\ E_{yy} \end{array}).$$ (3)

Here, |J⟩ is a polarization state described by the non-normalized Jones vector. We emphasize that expression Eq. (3) implies that an arbitrarily polarized electromagnetic field can be considered as a superposition of two linearly polarized fields R(|J⟩) and I(|J⟩) containing information on the phase difference $\delta =\delta_{y}-\delta_{x}$ between them. Jones vectors for all of the pure polarization states^42^^,^^43^ can be represented in this manner. In particular, for linear polarized light along x axis |H⟩ and along y axis |V⟩, we have

$$|H\rangle =(\begin{array}{c} 1\\ 0 \end{array})=(\begin{array}{c} 1\\ 0 \end{array})+j(\begin{array}{c} 0\\ 0 \end{array}),\;|V\rangle =(\begin{array}{c} 0\\ 1 \end{array})=(\begin{array}{c} 0\\ 1 \end{array})+j(\begin{array}{c} 0\\ 0 \end{array}).$$

Here, $\delta_{x}=\delta_{y}=0$. It is possible to write down both linear polarization vectors with account for non-zero phase shifts. For example, in case $\delta_{x}=\delta_{y}=\pi /4$:

$$|H\rangle =(\begin{array}{c} 1+j\\ 0 \end{array})=(\begin{array}{c} 1\\ 0 \end{array})+j(\begin{array}{c} 1\\ 0 \end{array}),\;|V\rangle =(\begin{array}{c} 0\\ 1+j \end{array})=(\begin{array}{c} 0\\ 1 \end{array})+j(\begin{array}{c} 0\\ 1 \end{array}).$$

Similarly, linearly polarized light components along diagonal directions can be expressed as

$$|L_{+45\;\deg}\rangle =(\begin{array}{c} 1\\ 1 \end{array})=(\begin{array}{c} 1\\ 1 \end{array})+j(\begin{array}{c} 0\\ 0 \end{array}),\;|L_{-45\;\deg}\rangle =(\begin{array}{c} 1\\ -1 \end{array})=(\begin{array}{c} 1\\ -1 \end{array})+j(\begin{array}{c} 0\\ 0 \end{array}).$$

In the following, we will mostly consider Jones vectors for the right circular polarization (RCP) and left circular polarization (LCP):

$$|\mathrm{RCP}\rangle =(\begin{array}{c} 1\\ j \end{array})=(\begin{array}{c} 1\\ 0 \end{array})+j(\begin{array}{c} 0\\ 1 \end{array}),\;|\mathrm{LCP}\rangle =(\begin{array}{c} j\\ 1 \end{array})=(\begin{array}{c} 0\\ 1 \end{array})+j(\begin{array}{c} 1\\ 0 \end{array}).$$ (4)

By substituting field components Eqs. (2) and (3) into the definition Eq. (1) and performing some straightforward algebra, we arrive at the following expressions for the Stokes vector:

$$(\begin{array}{c} I_{p}\\ Q_{p}\\ U_{p}\\ V_{p} \end{array})=\frac{1}{2}(\begin{array}{c} \left(E_{xx}^{2}+E_{yx}^{2})+(E_{xy}^{2}+E_{yy}^{2}\right)\\ \left(E_{xx}^{2}+E_{yx}^{2})-(E_{xy}^{2}+E_{yy}^{2}\right)\\ 2(E_{xx}E_{xy}+E_{yx}E_{yy})\\ 2(E_{xx}E_{yy}-E_{yx}E_{xy}) \end{array}).$$ (5)

It is important to note that here all variables are real-valued and that E components are in fact parts of the real-valued linearly polarized e/m waves R(|J⟩), I(|J⟩).

Established relation Eq. (5) is the fundamental one to relate Stokes formalism with the existing BS technique developed to trace evolution of Jones polarization vector along MC-photon trajectories.^13^^,^^19^^,^^47^ Stokes formalism enables to immediately recognize the CPL helicity flips appearing as the Stokes vector locus crossing equator on the Poincaré sphere [see Fig. 1(b)]. We note that Eqs. (1)–(5) are written in the local reference frame of the wave.

### 2.2 Degrees of Polarization

In order to consider partially polarized light field averaging procedures are commonly used. This can clearly be seen on the example of the Wolf’s coherence matrix J:^48^

$$J=(\begin{array}{cc} J_{xx} & J_{xy}\\ J_{yx} & J_{yy} \end{array})=(\begin{array}{cc} \left\langle E_{x}E_{x}^{*}\right\rangle & \left\langle E_{x}E_{y}^{*}\right\rangle \\ \left\langle E_{y}E_{x}^{*}\right\rangle & \left\langle E_{y}E_{y}^{*}\right\rangle \end{array})=\frac{1}{2}(\begin{array}{cc} Q+I & U+jV\\ U-jV & -Q+I \end{array}).$$ (6)

Here, $J_{xx}J_{yy}-J_{xy}J_{yx}\geq 0$. With Eq. (6) we have also provided a connection between coherence matrix and Stokes parameters (I,Q,U,V) of the partially polarized light. Brackets ⟨⟩ correspond to the field averaging procedure. Traditionally, time-averaging $\langle F(t)\rangle =\underset{T\to \infty }{\lim}\frac{1}{2T}\int_{-\infty }^{\infty }F(t)dt$ with respect to the detector finite integration time T is performed, along with spectral and spatial averaging defined by the resolution of the detector.^42^^,^^48^ In this work, brackets ⟨⟩ correspond to the averaging of MC photon intensities. This approach will be covered in Sec. 3.3. For partially polarized light, following definitions^43^^,^^48^ for the degrees of polarization based on the coherence matrix and Stokes approaches are used:

$$\mathrm{DoP}=\sqrt{1-\frac{4\det(J)}{{\left(J_{xx}+J_{yy}\right)}^{2}}}=\frac{\sqrt{Q^{2}+U^{2}+V^{3}}}{I},$$ (7)

$$\mathrm{DoLP}=\frac{\sqrt{{\left(J_{xx}-J_{yy}\right)}^{2}+{\left(J_{xy}+J_{yx}\right)}^{2}}}{J_{xx}+J_{yy}}=\frac{\sqrt{Q^{2}+U^{2}}}{I},$$ (8)

$$\mathrm{DoCP}=\frac{\sqrt{2J_{yx}J_{xy}-J_{yx}^{2}-J_{xy}^{2}}}{J_{xx}+J_{yy}}=\frac{\sqrt{V^{2}}}{I}.$$ (9)

Here, DoP is the total degree of polarization, DoLP is the degree of linear polarization, and DoCP is the degree of circular polarization, ${\mathrm{DoP}}^{2}={\mathrm{DoLP}}^{2}{+\mathrm{DoCP}}^{2}$. Partially polarized light can be decomposed into fully polarized and non-polarized parts:^43^

$$\begin{array}{c} (\begin{array}{c} I\\ Q\\ U\\ V \end{array})=(1-\mathrm{DoP})(\begin{array}{c} I\\ 0\\ 0\\ 0 \end{array})+(\begin{array}{c} \mathrm{DoP}\cdot I\\ Q\\ U\\ V \end{array}), \end{array}\;0\leq \mathrm{DoP}\leq 1.$$ (10)

Or, alternatively, partially polarized light can be treated as a superposition of two oppositely polarized waves:^43^

$$(\begin{array}{c} I\\ Q\\ U\\ V \end{array})=\frac{\left(1+\mathrm{DoP}\right)}{2\mathrm{DoP}}(\begin{array}{c} \mathrm{DoP}\cdot I\\ Q\\ U\\ V \end{array})+\frac{\left(1-\mathrm{DoP}\right)}{2\mathrm{DoP}}(\begin{array}{c} \mathrm{DoP}\cdot I\\ -Q\\ -U\\ -V \end{array}),\;0<\mathrm{DoP}\leq 1.$$ (11)

These expressions can be rewritten in more compact form by using Stokes parameters normalized to the intensity of the fully polarized component:

$$Q_{n}=\frac{Q}{\mathrm{DoP}\cdot I},\;U_{n}=\frac{U}{\mathrm{DoP}\cdot I},\;V_{n}=\frac{V}{\mathrm{DoP}\cdot I}.$$ (12)

This definition allows to compute the Stokes vector values that are typically provided, e.g., by ThorLabs polarimeters.^49^ In addition, we can assume that $Q_{n}=U_{n}=V_{n}=0$ when DoP=0 (all Stokes components of the fully depolarized part are equal to zero). Then Eq. (10) takes the form

$$(\begin{array}{c} I\\ Q\\ U\\ V \end{array})=(1-\mathrm{DoP})I(\begin{array}{c} 1\\ 0\\ 0\\ 0 \end{array})+\mathrm{DoP}\cdot I(\begin{array}{c} 1\\ Q_{n}\\ U_{n}\\ V_{n} \end{array}),$$ (13)

and Eq. (11) is written as

$$(\begin{array}{c} I\\ Q\\ U\\ V \end{array})=\frac{(1+\mathrm{DoP})I}{2}(\begin{array}{c} 1\\ Q_{n}\\ U_{n}\\ V_{n} \end{array})+\frac{(1-\mathrm{DoP})I}{2}(\begin{array}{c} 1\\ -Q_{n}\\ -U_{n}\\ -V_{n} \end{array}).$$ (14)

Now in both equations 0≤DoP≤1.

Important specific cases of the expressions Eqs. (13) and (14) include decomposition of the CPL into the fully polarized right- and left-handed parts and decomposition of the linearly polarized light into orthogonal components. For the first case, we rewrite Eq. (13) as

$$(\begin{array}{c} I\\ 0\\ 0\\ V \end{array})=(1-\mathrm{DoCP})I(\frac{1}{2}(\begin{array}{c} 1\\ 0\\ 0\\ -1 \end{array})+\frac{1}{2}(\begin{array}{c} 1\\ 0\\ 0\\ 1 \end{array}))+\mathrm{DoCP}\cdot I(\begin{array}{c} 1\\ 0\\ 0\\ 1 \end{array}),$$

after terms regroup arriving at

$$(\begin{array}{c} I\\ 0\\ 0\\ V \end{array})=\frac{(1-\mathrm{DoCP})I}{2}(\begin{array}{c} 1\\ 0\\ 0\\ -1 \end{array})+\frac{(1+\mathrm{DoCP})I}{2}(\begin{array}{c} 1\\ 0\\ 0\\ 1 \end{array}).$$ (15)

This alternative form of the expression Eq. (14) allows to write down expressions for the co- and cross-polarized light components via DoCP:

$$I_{R}=\frac{1}{2}(1+\mathrm{DoCP})S_{0},\;I_{L}=\frac{1}{2}(1-\mathrm{DoCP})S_{0}.$$

Here, $I_{R}$ corresponds to the RCP light and $I_{L}$ corresponds to the LCP light. DoCP value can then be estimated as

$$\mathrm{DoCP}=\frac{I_{R}-I_{L}}{I_{R}+I_{L}}.$$ (16)

We note that this expression has to be treated with care: when $I_{L}>I_{R}$, we supposedly arrive at negative DoCP values. However, this does not actually contradict the definition Eq. (9), because expression Eq. (16) is derived under the assumption that RCP intensity is always larger than LCP one, as follows from Eq. (15). Otherwise, we should appropriately rewrite these equations, arriving at $\mathrm{DoCP}=(I_{L}-I_{R})/(I_{L}+I_{R})$, which generally results in $\mathrm{DoCP}=|I_{R}-I_{L}|/(I_{R}+I_{L})$ fully complying with Eq. (9).

Similar decomposition can be written for the second case when light is linearly polarized:

$$(\begin{array}{c} I\\ Q\\ U\\ 0 \end{array})=\frac{(1+\mathrm{DoLP})I}{2}(\begin{array}{c} 1\\ Q_{n}\\ U_{n}\\ 0 \end{array})+\frac{(1-\mathrm{DoLP})I}{2}(\begin{array}{c} 1\\ -Q_{n}\\ -U_{n}\\ 0 \end{array}),$$ (17)

which in turn reduces to

$$(\begin{array}{c} I\\ Q\\ 0\\ 0 \end{array})=\frac{(1+DR)I}{2}(\begin{array}{c} 1\\ 1\\ 0\\ 0 \end{array})+\frac{(1-DR)I}{2}(\begin{array}{c} 1\\ -1\\ 0\\ 0 \end{array}),$$ (18)

when U=0. Here, DR=|Q|/I and all polarization degrees are within [0,1] limits. Intensities of horizontally $I_{∥}$ and vertically $I_{⊥}$ polarized light can be obtained from Eq. (18) to express $DR=\frac{I_{∥}-I_{⊥}}{I_{∥}+I_{⊥}}$. This expression for DR has been used throughout most of the previous works.^13^ DoLP also involves intensities of light linearly polarized along +45  deg and −45  deg axes:^43^

$$\mathrm{DoLP}=\frac{\sqrt{{\left(I_{∥}-I_{⊥}\right)}^{2}+{\left(I_{+45\;\deg}-I_{-45\;\deg}\right)}^{2}}}{I}.$$ (19)

Here, $I=I_{∥}+I_{⊥}=I_{+45\;\deg}+I_{-45\;\deg}=I_{R}+I_{L}$. Now, we have established theoretical background and can proceed with the description of the developed MC approach.

## 3 Monte Carlo Based on the Bethe–Salpeter Equation

### 3.1 Tracking of the Jones Polarization Vector

**Fig. 2:**
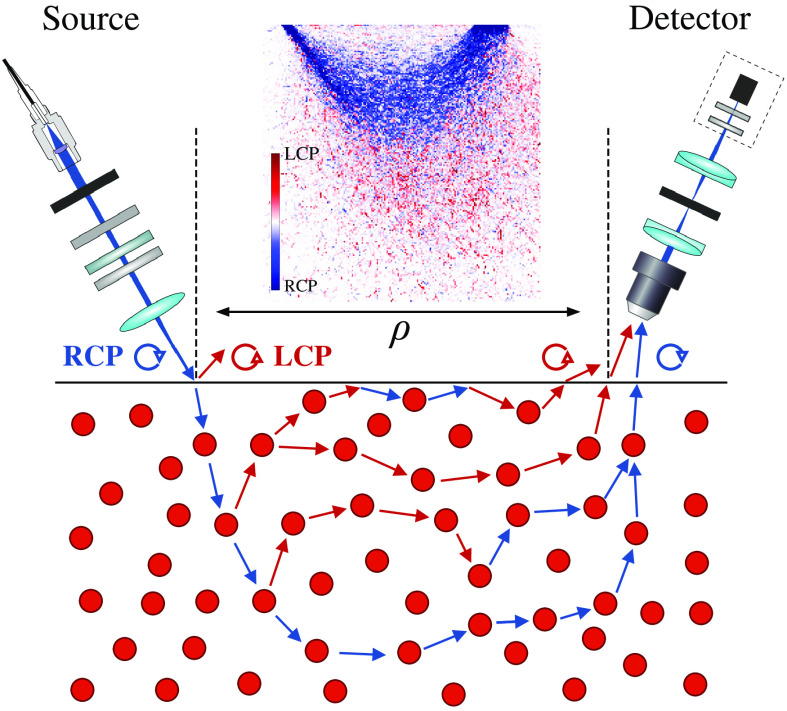
Illustration of the backscattering model with schematically depicted elements of the experimental setup.^4^^–^^6^ Sample with known optical properties is illuminated with RCP light. Possible MC-photon trajectories with zero, one, and two backscattering events and with photon-surface interactions are presented. Each backscattering event causes a helicity flip represented by the color of the direction arrow. The experimental configuration involves supercontinuum fiber laser source filtered by the acousto-optic tunable filter. The resulting RCP is produced with the half-wave and quarter-wave plates and is focused on the medium surface under $\theta_{i}$ angle. The detector is oriented under $-\theta_{d}$ angle to the surface normal, collects backscattered light with 20× objective lens and measures Stokes parameters of the registered light with a polarimeter.^49^ The inset shows simulated sampling volumes for RCP and LCP light components at the relatively large source–detector separation distance ρ (see Sec. 4.3 for more details).

Within the BS-based MC model,^13^^,^^19^^,^^33^ a large amount ($N_{\mathrm{inc}}>10^{9}$) of MC-photons with pre-defined statistical weight $W_{j}$, $j=[1...N_{\mathrm{inc}}]$ is launched from the source oriented under $\theta_{i}$ angle to the surface normal, propagates through the turbid medium and statistics is collected from those $N_{\mathrm{ph}}<N_{\mathrm{inc}}$ arrived on the detector oriented under $-\theta_{d}$ angle to the surface normal (see Fig. 2). Here, the minus sign corresponds to the opposite direction of the detector to the surface normal as compared with the direction of the source. Turbid medium is defined by scattering coefficient $\mu_{s}$, absorption coefficient $\mu_{a}$, anisotropy parameter g, and refractive index n.^18^ In addition, tissue-like medium implies low contrast between refractive indices of the host medium and scatterers (e.g., cellular components, organelles, extracellular matrices, and other microstructures).

In this work, we consider a uniform distribution of MC-photons, noting that in general our approach allows to simulate spatial and phase distributions for a wide variety of light beams, including Gaussian, Bessel, Hermite–Gaussian, and Laguerre–Gaussian beams with complex shape carrying orbital angular momentum (OAM). To account for these beam types, it is necessary both to ensure the appropriate initial distribution of the MC-photons relevant to the beam intensity and phase profiles and to set the correct initial directions of the MC-photons according to the Poynting vector trajectories that render energy transfer within the beam.^50^^,^^51^ With the next development, we plan to implement this technique in our model to investigate the conservation of OAM in tissue-like medium.

Each MC-photon at the source is characterized by the initial statistical weight $W_{0_{j}}$, Cartesian coordinates $\left(x_{0},y_{0},0\right)$, propagation direction $s_{0}$ (defined both by beam structure and angle $\theta_{i}$ between source and surface normal, see Fig. 2) and, most importantly, by the initial polarization state. We introduce a real-valued vector P that corresponds to the direction of the linearly polarized E field.^13^^,^^19^^,^^32^^–^^34^^,^^39^ By assigning a pair of these vectors $P_{x}=(P_{xx},P_{xy},P_{xz})$, $P_{y}=(P_{yx},P_{yy},P_{yz})$ to each MC-photon, we are able to define two separate independent linear polarization states similarly to Eq. (3). It is important to note that here both polarization vectors are written in the global Cartesian coordinate system (x,y,z) and that they are orthogonal to the MC-photon unit propagation direction. If photon direction coincides with the z axis, then sum of $P_{x}∼R(|J\rangle )$ and $P_{y}∼I(|J\rangle )$ can be interpreted as Jones vector: $|J\rangle =P_{x}+jP_{y}$. We emphasize that from $P_{x}$ and $P_{y}$ we can always compute the Jones vector associated with the MC-photon and vice versa; by knowing the polarization state (Jones vector) of the MC-photon we can always reconstruct $P_{x}$ and $P_{y}$ values.

After launch, all MC-photons undergo surface (z=0) interaction and are transmitted to the turbid medium layer with account for the Snell’s law and the appropriate Fresnel coefficients influencing MC-photon weights, directions, and polarization (see Sec. 3.2). In turbid medium (z>0) each MC-photon trajectory is modeled as a sequence of the elementary simulations containing limited amount of scattering events $N_{\text{scatt}}$. This procedure has been thoroughly covered in the previous works.^13^^,^^19^^,^^47^ At each i’th scattering event, $i=[1...N_{\text{scatt}}]$, the following computational steps are performed: random path length $l_{i}=-\ln\;\xi /\mu_{s}$ is computed (in this paper, we assume that $\mu_{a}\ll \mu_{s}$ and ξ∈(0,1] is a uniformly distributed random number), MC-photon is moved to the next position $r_{i}=r_{i-1}+s_{i}l_{i}$ with weight attenuated according to the Beer–Lambert law ($W_{i}=W_{i-1}e^{-\mu_{a}l_{i}}$), and the next propagation direction $s_{i+1}$ is evaluated via inversion of the Henyey–Greenstein (HG) phase function^52^

$$p_{\mathrm{HG}}(\cos\;\theta^{\prime })=\frac{1}{4\pi }\frac{1-g^{2}}{{\left(1+g^{2}-2g\;\cos\;\theta^{\prime }\right)}^{3/2}},$$

where $\theta^{\prime }$ is the polar scattering angle in the MC-photon reference plane. Here, we have used the position vector $r_{i}=(x_{i},y_{i},z_{i})$ and the unit direction for each scattering event $s_{i}=[s_{X},s_{Y},s_{Z}{]}_{i}=[\sin\;\theta \;\cos\;\varphi ,\;\sin\;\theta \;\sin\;\varphi ,\;\cos\;\theta {]}_{i}$, with θ,φ as azimuthal and polar angles that correspond to the global Cartesian coordinates. HG function has been traditionally employed in the MC simulations as a substitute to the rigorous Mie phase function due to its high performance and the ability to provide realistic results complying with the experimental tissue measurements.^15^^,^^53^^,^^54^ It should be noted that, basically, any phase function p can be used.^55^^,^^56^ If analytical inversion of p is not possible, e.g., for the case of Mie scattering, then table lookup method is involved to ensure fast computational speed.

At each step, we check if MC-photon path crosses the medium boundary and invoke surface refraction–transmission and detection procedures if this is the case (see Sec. 3.2). Evolution of each linearly polarized state $P_{x},P_{y}$ can be traced along the MC-photon trajectory $r_{i},i=[1...N_{\text{scatt}}]$ via the following procedure, which is obtained from the iterative solution to BS equation:^13^^,^^14^^,^^19^

Pi=−si×[si×Pi−1]=[I^−si⊗si]⏟U^iPi−1. (20)

Here, $\hat{I}$ is the third-rank unit tensor and ⊗ indicates the direct product. Tensor $\left[\hat{I}-s_{i}⊗s_{i}\right]$ can be explicitly rewritten in the form of 3×3 real operator U^i:^32^

U^i=(1−siX2−siX·siY−siX·siZ−siX·siY1−siY2−siY·siZ−siX·siZ−siX·siZ1−siZ2).

Most importantly, operator U^i guarantees that the electromagnetic field remains transversal experiencing the i’th scattering event. It can be applied to both linear polarization vectors $P_{x},P_{y}$ simultaneously as follows from Eq. (2), and it accounts for the helicity flips when considering pair of the polarization vectors that correspond to the circularly or elliptically polarized MC-photon [see Fig. 1(a)]. Eventually, the chain U^NU^N−1U^N−2…U^2U^1 of projection operators transforms the initial polarization $P_{x_{0}}$ upon a sequence of N scattering events to the final polarization $P_{x_{N}}$:^19^

PxN=U^NU^N−1U^N−2…U^1Px0. (21)

The same expression can be used to relate $P_{y_{N}}$ and $P_{y_{0}}$ as follows from Eq. (2). It is important to note that this procedure always ensures $P_{x_{i}}$ and $P_{y_{i}}$ to be orthogonal to the MC-photon direction $s_{i}$ at each scattering event. It means that if we rewrite $P_{x_{i}}$ and $P_{y_{i}}$ in terms of the MC-photon local reference frame using the appropriate transformation matrix, we will obtain Jones vectors with third component equal to zero. This peculiarity can be verified, e.g., numerically, but, most importantly, polarization tracing Eq. (21) does not inherently require reference frame tracking and allows one to avoid computation of the scattering and rotation matrices as proposed by the VRTE-based approaches,^23^^,^^28^ leading to the computational demand of polarization-enabled MC to be comparable to the demand of scalar MC. Tensor U^i ensures that each individual MC-photon always remains fully polarized. Then Stokes vector values can be obtained for each MC-photon at any scattering event via Eq. (5) with E values replaced by the corresponding $P_{x_{i}},P_{y_{i}}$ components.

We should explicitly note that the approach based on the Bethe–Salpeter equation was rigorously introduced for the case of pure Rayleigh scattering.^32^ In case of biotissues, we deal with scatterers with the size comparable to or a few times higher than the wavelength λ. Keeping in mind that within biological media fluctuation of the relative refractive index $n_{r}$ between the scatterer (e.g., cell component such as nucleus, $n_{s}$) and the surrounding medium (e.g., cytoplasm, $n_{m}$) is typically small ($|n_{r}-1|<0.1$, $n_{r}=n_{s}/n_{m}$),^18^ we conclude that we actually deal with the so-called soft scattering particles.^57^^,^^58^ In this case, particle size d should obey the relation $kd|n_{r}-1|\ll 1$, where k=2π/λ. Then Rayleigh–Gans–Debye (RGD) approach can be applied to describe scattering by soft particles characterized by the non-isotropic scattering phase function.^32^^,^^57^^,^^58^ On these grounds, the proposed BS-based MC polarization tracing can be treated as the first-order approximation to RGD and applied to simulate polarized light scattering in biological media.^19^^,^^32^ We also note that in this paper non-birefringent and non-optically active medium is considered; while birefringence is known to be an important feature of biological tissues, it has been reported that, e.g., for skin it is almost impossible to observe the phase changes occurring due to birefringence at normal conditions.^59^ At the same time, account for birefringence can be added into the developed model by properly implementing account for the ordinary and extraordinary optical pathlengths of MC-photons influencing the phase shift and polarization state.

We repeat the outlined computational steps for each scattering event until one of the following conditions is met: either $W_{i}<10^{-4}$ (statistical weight becomes negligible as follows from the Beer–Lambert law) or the amount of scattering events $N_{\text{scatt}}$ becomes larger than $10^{3}$. These limitations ensure proper trajectory tracing cut-off.^19^ We continue launching MC-photons until the certain amount (no less than $N_{\mathrm{ph}}=10^{5}$) arrives on the detector. Detection procedure consists of the two checks: MC-photon coordinates should lie within the detector area ($-r_{d}+\rho \leq x_{N}\leq r_{d}+\rho$, $-r_{d}\leq y_{N}\leq r_{d},z_{N}=0$) and refracted direction $s_{N}$ should meet the detector numerical aperture (NA) requirements. We would limit those directions by using $\mathrm{acos}(s_{N}\cdot s_{d})<\mathrm{NA}$, where $s_{d}=[\sin(-\theta_{d}),0,\cos(-\theta_{d})]$ is the unit vector collinear to the detector axis. Both here and in the subsequent sections, N is considered to be an index of the detection event.

### 3.2 Interface Influence

Operator U^i allows us to trace the polarization evolution at each scattering event within the turbid medium, as shown by Eq. (21). However, it does not account for the phenomena occurring at the medium boundaries. In this case, the well-known Fresnel coefficients have to be applied to polarized light:^48^ $T_{P}=\frac{2n_{1}\;\cos\;\theta_{c}}{n_{2}\;\cos\;\theta_{c}+n_{1}\;\cos\;\theta_{t}}$, $T_{S}=\frac{2n_{1}\;\cos\;\theta_{c}}{n_{1}\;\cos\;\theta_{c}+n_{2}\;\cos\;\theta_{t}}$, $R_{P}=\frac{n_{2}\;\cos\;\theta_{c}-n_{1}\;\cos\;\theta_{c}}{n_{2}\;\cos\;\theta_{c}+n_{1}\;\cos\;\theta_{t}}$, $R_{S}=\frac{n_{1}\;\cos\;\theta_{c}-n_{2}\;\cos\;\theta_{t}}{n_{1}\;\cos\;\theta_{c}+n_{2}\;\cos\;\theta_{t}}$. Here, $T_{P},T_{S}$ correspond to the transmission coefficients for P- and S-polarized (or |H⟩ and |V⟩) waves, and $R_{P},R_{S}$ correspond to the reflection coefficients. We have also introduced angle of the incident light $\theta_{c}$, angle of the transmitted light $\theta_{t}$, and medium refractive indices $n_{1,2}$. Fresnel coefficients can be complex-valued, for example, in case of total internal reflection due to Snell law $n_{1}\;\sin\;\theta_{c}=n_{2}\;\sin\;\theta_{t}$. As a consequence, these coefficients cannot be separately applied to each linear polarization vector $P_{x,y}$; instead, the complex counterpart of Eq. (3) has to be reconstructed from the pair of vectors Eq. (2) prior to applying Fresnel coefficients. After that, the new reflected or transmitted vectors can be decomposed back into two separate linear polarization states, and polarization tracing procedure from Sec. 3.1 can be continued. We also have to keep in mind that Fresnel coefficients are derived in the wave’s plane of incidence.^48^ It means that at the event of the MC-photon interaction with the surface we have to rewrite both P vectors in the corresponding reference frame $\left(x^{\prime },y^{\prime },z^{\prime }\right)$, defined by the MC-photon direction and its projection to the interface of the surface, via applying proper transformation matrix.

If i−1 is the index of the event of the MC-photon interaction with the surface, and i is the index of the next scattering event, account for the Fresnel coefficients can be mathematically expressed in the following form: ${\left(P_{x}^{\prime }\right)}_{i}={\left(P_{x}^{\prime }\right)}_{i-1}\cdot R_{P}$, ${\left(P_{y}^{\prime }\right)}_{i}={\left(P_{y}^{\prime }\right)}_{i-1}\cdot R_{S}$, ${\left(P_{z}^{\prime }\right)}_{i}={\left(P_{z}^{\prime }\right)}_{i-1}\cdot R_{P}$. Here, $P^{\prime }$ are polarization vectors transformed to the reference frame associated with the MC-photon’s plane of incidence. In terms of polarization vector components:

$$(P_{xx}^{\prime }{)}_{i}=R(R_{P}){\left(P_{xx}^{\prime }\right)}_{i-1}-I(R_{P}){\left(P_{yx}^{\prime }\right)}_{i-1},\;(P_{yx}^{\prime }{)}_{i}=I(R_{P}){\left(P_{xx}^{\prime }\right)}_{i-1}+R(R_{P}){\left(P_{yx}^{\prime }\right)}_{i-1}\;(P_{xy}^{\prime }{)}_{i}=R(R_{S}){\left(P_{xy}^{\prime }\right)}_{i-1}-I(R_{S}){\left(P_{yy}^{\prime }\right)}_{i-1},\;(P_{yy}^{\prime }{)}_{i}=I(R_{S}){\left(P_{xy}^{\prime }\right)}_{i-1}+R(R_{S}){\left(P_{yy}^{\prime }\right)}_{i-1},\;(P_{xz}^{\prime }{)}_{i}=R(R_{P}){\left(P_{xz}^{\prime }\right)}_{i-1}-I(R_{P}){\left(P_{yz}^{\prime }\right)}_{i-1},\;(P_{yz}^{\prime }{)}_{i}=I(R_{P}){\left(P_{xz}^{\prime }\right)}_{i-1}+R(R_{P}){\left(P_{yz}^{\prime }\right)}_{i-1}.$$ (22)

For the transmission, it is enough to replace $R_{P},R_{S}$ with their counterparts $T_{P},T_{S}$. At the same time, in the specific case of linearly polarized light where phase information is not usually relevant, the field has only one polarization vector $P_{x}$, and it is possible to account for polarization changes at the interface via absolute values $|T_{P}{|}^{2},|T_{S}{|}^{2},|R_{P}{|}^{2},|R_{S}{|}^{2}$ of Fresnel coefficients as outlined in the previous works.^13^ This procedure influences the absolute value of polarization vectors, and, correspondingly, the weight of each MC-photon. After account for the interface influence, both $P^{\prime }$ vectors are transformed back to the global (x,y,z) reference frame. We would further use the notations $\left(x^{\prime },y^{\prime },z^{\prime }\right)$ and $P^{\prime }$ in order to emphasize that non-laboratory reference frame is used; in addition to the plane of incidence, this could be either source or detector reference frame, or local reference frame of the MC-photon.

We also note that it is necessary to properly select transmitted or reflected MC-photons in multilayered medium. It can be done via implementing selection procedure following Wang^15^ at each interface between medium layers, adding proper account for the polarization state of the MC-photon. In this work, we consider homogeneous scattering medium with single layer.

### 3.3 Detected Light Intensity Components, Stokes Vector, and Polarization Degrees

Each MC-photon that arrived on the detector is fully polarized and its polarization state is known from Eq. (21) with account for reflections/refractions by Eq. (22). Every detected MC-photon also possesses weight attenuated with respect to the Beer–Lambert law $W_{N_{j}}=W_{0_{j}}\;\exp(-\mu_{a}\overset{N_{j}}{\underset{i=1}{\sum }}l_{i})$, where $0<N_{j}<N_{\text{scatt}}$ is index of the detection event for j’th MC-photon, and $l_{i}$ is the path length between two neighboring scattering events. If detector plane is parallel to the medium surface, then averaging of the MC-photon ensemble intensity components is performed as follows:^34^^,^^39^

$$I_{R}=\frac{1}{4N_{\mathrm{inc}}}\overset{N_{\mathrm{ph}}}{\underset{j=1}{\sum }}W_{N_{j}}(P_{xx}^{2}+P_{yx}^{2}+P_{xy}^{2}+P_{yy}^{2}+2P_{xx}P_{yy}-2P_{yx}P_{xy}{)}_{N_{j}}\Gamma_{R}^{N_{j}},$$ (23)

$$I_{L}=\frac{1}{4N_{\mathrm{inc}}}\overset{N_{\mathrm{ph}}}{\underset{j=1}{\sum }}W_{N_{j}}{\left(P_{xx}^{2}+P_{yx}^{2}+P_{xy}^{2}+P_{yy}^{2}-2P_{xx}P_{yy}+2P_{yx}P_{xy}\right)}_{N_{j}}\Gamma_{R}^{N_{j}}.$$ (24)

For completeness, we also provide expressions for all intensities of the linearly polarized light:

$$I_{+45\;\deg}=\frac{1}{4N_{\mathrm{inc}}}\overset{N_{\mathrm{ph}}}{\underset{j=1}{\sum }}W_{N_{j}}(P_{xx}^{2}+P_{yx}^{2}+P_{xy}^{2}+P_{yy}^{2}+2P_{xx}P_{xy}+2P_{yx}P_{yy}{)}_{N_{j}}\Gamma_{R}^{N_{j}},$$ (25)

$$I_{-45\;\deg}=\frac{1}{4N_{\mathrm{inc}}}\overset{N_{\mathrm{ph}}}{\underset{j=1}{\sum }}W_{N_{j}}{\left(P_{xx}^{2}+P_{yx}^{2}+P_{xy}^{2}+P_{yy}^{2}-2P_{xx}P_{xy}-2P_{yx}P_{yy}\right)}_{N_{j}}\Gamma_{R}^{N_{j}},$$ (26)

$$I_{∥}=\frac{1}{2N_{\mathrm{inc}}}\overset{N_{\mathrm{ph}}}{\underset{j=1}{\sum }}W_{N_{j}}(P_{xx}^{2}+P_{yx}^{2}{)}_{N_{j}}\Gamma_{R}^{N_{j}},$$ (27)

$$I_{⊥}=\frac{1}{2N_{\mathrm{inc}}}\overset{N_{\mathrm{ph}}}{\underset{j=1}{\sum }}W_{N_{j}}(P_{xy}^{2}+P_{yy}^{2}{)}_{N_{j}}\Gamma_{R}^{N_{j}}.$$ (28)

Here, $\Gamma_{R}=\frac{2}{1+\overset{¯}{\cos^{2}\;\theta }}$ is the Rayleigh factor derived from the optical theorem in Born approximation and $\overset{¯}{\cos^{2}\;\theta }$ is the square cosine of the scattering angle weighted by the single scattering cross-section. ^13^^,^^19^^,^^32^^,^^33^ For an arbitrary orientation of the detector (see Fig. 2), both $P_{x}$ and $P_{y}$ are supposed to be rewritten in the new Cartesian basis with $z^{\prime }$ axis being collinear to the detector axis.

Stokes parameters are related to the light intensity components as

$$Q=I_{∥}-I_{⊥},\;U=I_{+45\;\deg}-I_{-45\;\deg},\;V=I_{R}-I_{L}.$$ (29)

Final expressions for the Stokes parameters withing the BS-based MC are

$$I=\frac{1}{2N_{\mathrm{inc}}}\overset{N_{\mathrm{ph}}}{\underset{j=1}{\sum }}W_{N_{j}}(P_{xx}^{2}+P_{yx}^{2}+P_{xy}^{2}+P_{yy}^{2}{)}_{N_{j}}\Gamma_{R}^{N_{j}},$$ (30a)

$$Q=\frac{1}{2N_{\mathrm{inc}}}\sum_{j=1}^{N_{\mathrm{ph}}}W_{N_{j}}(P_{xx}^{2}+P_{yx}^{2}-P_{xy}^{2}-P_{yy}^{2}{)}_{N_{j}}\Gamma_{R}^{N_{j}},$$ (30b)

$$U=\frac{1}{N_{\mathrm{inc}}}\sum_{j=1}^{N_{\mathrm{ph}}}W_{N_{j}}(P_{xx}P_{xy}+P_{yx}P_{yy}{)}_{N_{j}}\Gamma_{R}^{N_{j}},$$ (30c)

$$V=\frac{1}{N_{\mathrm{inc}}}\overset{N_{\mathrm{ph}}}{\underset{j=1}{\sum }}W_{N_{j}}(P_{xx}P_{yy}-P_{yx}P_{xy}{)}_{N_{j}}\Gamma_{R}^{N_{j}}.$$ (30d)

Degrees of polarization can then be computed either via definitions Eqs. (7)–(9) or, equivalently, via expressions for intensity components Eqs. (16) and (19). Depending on the detection conditions, it might be necessary to compute any of the given parameters in the reference frame other than the global one, e.g., in the detector reference frame or in the local reference frame of each MC-photon. For this purpose, transformation matrix providing $P^{\prime }$ in the selected reference frame $\left(x^{\prime },y^{\prime },z^{\prime }\right)$ can be used. The obtained $P^{\prime }$ values can be directly substituted into Eqs. (23)–(30) providing appropriate intensity, Stokes, or degree of polarization values.

### 3.4 Computation of Mueller Matrix Components

We have demonstrated that within the proposed MC approach, such parameters as Jones vector Eq. (21), Stokes vector for partially polarized light Eq. (30), Wolf coherence matrix Eq. (6), and degrees of polarization Eqs. (7)–(9), can be evaluated. We also stress that it is possible to compute Mueller matrix elements. We consider Mueller matrix in its general form:

$$M=[\begin{array}{cccc} m_{11} & m_{12} & m_{13} & m_{14}\\ m_{21} & m_{22} & m_{23} & m_{24}\\ m_{31} & m_{32} & m_{33} & m_{34}\\ m_{41} & m_{42} & m_{43} & m_{44} \end{array}],\;{\left(\begin{array}{c} I\\ Q\\ U\\ V \end{array}\right)}_{\text{out}}=M{\left(\begin{array}{c} I\\ Q\\ U\\ V \end{array}\right)}_{\text{in}}.$$

Mueller matrix elements are usually measured with the following setup configurations:^60^

$$M=[\begin{array}{cccc} OO & HO-VO & PO-MO & LO-RO\\ OH-OV & \left(HH+VV)-(HV+VH\right) & \left(PH+MV)-(PV+MH\right) & \left(LH+RV)-(LV+RH\right)\\ OP-OM & \left(HP+VM)-(HM+VP\right) & \left(PP+MM)-(PM+MP\right) & \left(LP+RM)-(LM+RP\right)\\ OL-OR & \left(HL+VR)-(HR+VL\right) & \left(PL+MR)-(PR+ML\right) & \left(LL+RR)-(RL+LR\right) \end{array}].$$ (31)

Here, the first letter corresponds to the source polarization, and the second letter corresponds to the measured intensity (with analyzer); O is the non-polarized light, H corresponds to $I_{∥}$, V to $I_{⊥}$, P to $I_{+45\;\deg}$, M to $I_{-45\;\deg}$, R to $I_{R}$, and L to $I_{L}$. In terms of our model, Mueller matrix M of the single detected photon can be expressed as

$$\begin{array}{llll} M_{11}=I & M_{12}=P_{xx}^{2}+P_{xy}^{2}-P_{yx}^{2}-P_{yy}^{2} & M_{13}=P_{xx}^{2}+P_{xy}^{2}-P_{yx}^{2}-P_{yy}^{2} & M_{14}=0\\ M_{21}=M_{12} & M_{22}=P_{xx}^{2}-P_{xy}^{2}-P_{yx}^{2}+P_{yy}^{2} & M_{23}=P_{xx}^{2}-P_{xy}^{2}-P_{yx}^{2}+P_{yy}^{2} & M_{24}=0\\ M_{31}=M_{12}^{rot} & M_{32}=P_{xx}P_{xy}-P_{yx}P_{yy} & M_{33}=P_{xx}P_{xy}-P_{yx}P_{yy} & M_{34}=0\\ M_{41}=M_{14} & M_{42}=0 & M_{43}=0 & M_{44}=P_{xx}P_{yy}-P_{xy}P_{yx} \end{array}.$$ (32)

Here, $P_{x}=(P_{xx},P_{xy},P_{xz})$ and $P_{y}=(P_{yx},P_{yy},P_{yz})$ are the real-valued vectors introduced in Sec. 3.1 and computed via Eq. (21) for incident linear polarizations $\left|H\rangle =P_{x_{0}}=(1,0,0\right)$, $\left|V\rangle =P_{y_{0}}=(0,1,0\right)$. Similarly, $P_{x}=(P_{xx},P_{xy},P_{xz})$ and $P_{y}=(P_{yx},P_{yy},P_{yz})$ are vectors computed for incident diagonal linear polarizations $\left|L_{+45\;\deg}\rangle =P_{x_{0}}=(\frac{\sqrt{2}}{2},\frac{\sqrt{2}}{2},0\right)$ and $\left|L_{-45\;\deg}\rangle =P_{y_{0}}=(\frac{\sqrt{2}}{2},-\frac{\sqrt{2}}{2},0\right)$. Circular polarization states |RCP⟩ and |LCP⟩ are accounted for as superpositions of |H⟩ and |V⟩ according to Eq. (4). $M_{31}=M_{12}^{\mathrm{rot}}$ means that this element can be obtained via rotation of $M_{12}$ by −π/4.^60^ Matrix Eq. (32) is valid when the detector plane coincides with the medium surface, as outlined in Sec. 3.3. Mueller matrix of the detected signal can then be obtained via the ensemble averaging procedure following the Eqs. (23)–(28):

$$M=\overset{N_{\mathrm{ph}}}{\underset{j=1}{\sum }}W_{N_{j}}M_{N_{j}}\Gamma_{R}^{N_{j}}.$$ (33)

Here, $M_{N_{j}}$ corresponds to the Mueller matrix of the j’th photon, which was detected at the $N_{j}$ scattering event, and all Mueller matrix elements are independently multiplied by the scalar term $W_{N_{j}}\Gamma_{R}^{N_{j}}$ for each photon. Now our formulation of the generalized BS-based polarization MC is complete. We emphasize that with Eqs. (32)–(33) we can compute Mueller matrix within one simulation, so it is not required to launch separate MC-photons with different polarization states. This factor, along with the remarks made in Sec. 3.1 [see Eq. (21)], contributes to the high computational performance of our approach.

## 4 Results and Discussion

### 4.1 Setup Configuration

Our theoretical model is oriented toward the most common experimental setups employed to study both forward (transmission) scattering and backscattering by biotissues with non-invasive diagnostic purposes.^61^ In particular, we verify the obtained simulation results against measurements performed with the backscattering setup, which has been thoroughly described in our previous works.^4^^–^^6^ In this setup, we employ multiwavelength 450 to 650 nm light source with 15  μm diameter incident under $\theta_{i}$ on the tissue-like surface characterized by $\mu_{s},\mu_{a},g$, and n. In the following, these values are selected to closely match the properties of real tissues or tissue phantoms.^62^ Incident light is right circularly polarized. We collect the scattered depolarized signal in the detector with 50  μm diameter oriented under $\theta_{d}$ with respect to surface normal and separated from the source by distance ρ (see Fig. 2). In order to properly study the evolution of CPL, we use an infinity-corrected objective in the detection arm ensuring that polarimeter registers Stokes parameters that correspond to the MC-photon local reference frames.

In this paper, incident |RCP⟩ beam is simulated as a plane wave (uniform distribution of MC-photons, direction defined solely by $\theta_{i}$) with λ=640  nm and polarization vectors defined as ${P^{\prime }}_{x_{0}}=(1,0,0)$, ${P^{\prime }}_{y_{0}}=(0,1,0)$ in the reference frame of the source. In the global reference frame, which is further employed in the scattering simulation, these vectors take the following form: $P_{x_{0}}=(\cos\;\theta_{i},0,\;\sin\;\theta_{i})$, $P_{y_{0}}=(0,1,0)$. In the model, we consider two source–detector configurations: with the angular incidence and collection of light ($\theta_{i}=55\;\deg$, $\theta_{d}=30\;\deg$), and with the vertically positioned source and detector ($\theta_{i}=\theta_{d}=0$). The ρ value is scaled to the transport mean free path $l^{*}=\mu_{s}^{-1}{\left(1-g\right)}^{-1}$ representing the average distance that light propagates before its direction of propagation is randomized.^58^^,^^63^^,^^64^ We collect detector statistics Eqs. (23)–(30) via evaluating polarization vectors in the local reference frame for each MC-photon, which corresponds to the experimental detection conditions.

### 4.2 Depolarization of the CPL Backscattered by Turbid Tissue-Like Medium

**Fig. 3:**
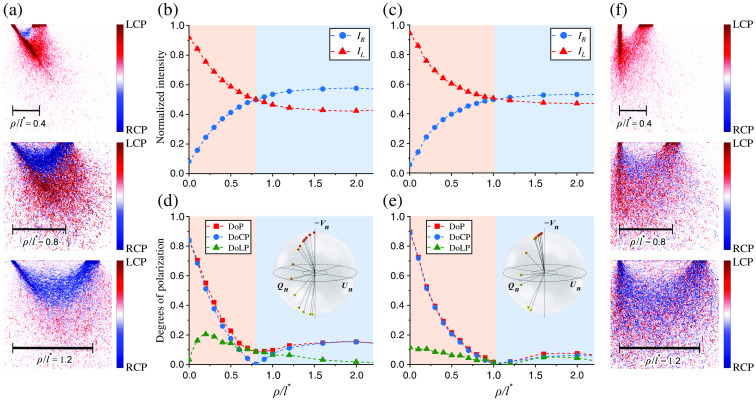
(a) Difference between sampling volumes for the intensity of cross-polarized $I_{L}$ (red) and co-polarized $I_{R}$ (blue) light arriving on the detector for the $\theta_{i}=55\;\deg$, $\theta_{d}=30\;\deg$ setup configuration with the variable source–detector separation distance ρ expressed in terms of transport length $l^{*}$, (b) $I_{L},I_{R}$ as functions of the source–detector separation for the $\theta_{i}=55\;\deg$, $\theta_{d}=30\;\deg$ setup, (c) the same for the $\theta_{i}=\theta_{d}=0\;\deg$ setup, (d) degrees of polarization DoP (red), DoCP (blue), DoLP (green), and corresponding normalized Stokes vector components $Q_{n},U_{n},V_{n}$ on the Poincaré sphere for the $\theta_{i}=55\;\deg$, $\theta_{d}=30\;\deg$ setup, (e) the same for the $\theta_{i}=\theta_{d}=0\;\deg$ setup, (f) difference between $I_{L},I_{R}$ sampling volumes for the $\theta_{i}=\theta_{d}=0\;\deg$ setup and the same source–detector separation distances $\rho /l^{*}$ as on (a). In these simulations detector with open NA has been considered. Points on the Poincaré spheres are colored gradually from red to yellow, which corresponds to the increase of $\rho /l^{*}$ distance.

We investigate the process of CPL depolarization in terms of the Stokes vector and light intensity components both via processing surface signal registered by the detector (see Sec. 3.1) and via analyzing in-depth distribution of the detected response represented by sampling volume.^16^^,^^17^ Main results are summarized in Fig. 3. We begin the analysis by studying the intensity components of the scattered light. Figures 3(b) and 3(c) show an interplay of the oppositely polarized RCP (blue) and LCP (red) intensities upon increase of the source–detector separation $\rho /l^{*}$. As one can see, for the short separation distances ($\rho /l^{*}<1$ for the vertical setup and $\rho /l^{*}<0.8$ for the angular setup), the helicity of incident RCP light is flipped due to backscattering, and the flipped LCP light is inversely related to the emerging RCP component. The LCP light is formed due to odd number of the helicity flips occurred along the consecutive scattering events within the medium between points of incidence and detection, whereas the appearance of RCP is based on the even number of flips.^44^ The decrease of LCP with the increase of source–detector separation is compensated with the proportional increase of RCP light, clearly illustrating predictions Eq. (15).

The RCP stream becomes dominating over the LCP at larger source–detector separation ($\rho >l^{*}$). This allows us to conclude that the angular momentum of light is preserved and that multiple scattering maintains the helicity of incident CPL (RCP). At the flip point (demarcated by red and blue background colors), the intensities of two streams of light with opposite helicities are equalized ($I_{R}=I_{L}$) and their superposition originates linear polarization. The polarization memory is revealed as a flip of the backscattered CPL at the source–detector separation over the transport length ($\rho >l^{*}$), tailing the helicity of incident RCP light. The resulting superposition of the scattered RCP and LCP light is registered by the detector as elliptically polarized light. It should be noted that elliptical polarization can be observed with any non-zero phase of the incident CPL if the plane of observation is not parallel or perpendicular to the original vibration direction of the field, which is accounted for in the developed model.

We proceed with the analysis of light depolarization by comparing DoP, DoLP, and DoCP versus source–detector separation. Corresponding plots are presented in Figs. 3(d) and 3(e) along with the normalized Stokes vector components $Q_{n},U_{n},V_{n}$ are depicted on the Poincaré sphere. DoCP represents the fraction of the CPL that is preserved or retained after the multiple scattering. With the increase of source–detector separation the DoCP is decreased due to reduction of low scattering orders contribution to the backscattered light. At a particular source–detector separation where flipped $I_{L}$ and preserved $I_{R}$ components of the backscattered CPL are equalized [see Figs. 2(b) and 2(c)], the DoCP reaches a minimum value. The depolarization minimum represents the point at which the components of scattered circularly light with opposite helicity, LCP and RCP, are superimposed. The depolarization minimum is coincided with the demarcation line between non-diffusive and diffusing path lengths of scattering photons characterized by $l^{*}$. This phenomenon is well pronounced when utilizing the angular source–detector configuration [see Figs. 2(b) and 2(d)]. These results significantly contribute to our understanding of the depolarization processes within tissues and prove to be useful, e.g., for the advanced alignment of the experimental setup with a conventional polarimeter employed to measure Stokes parameters and degrees of polarization of the backscattered elliptically polarized light.

**Fig. 4:**
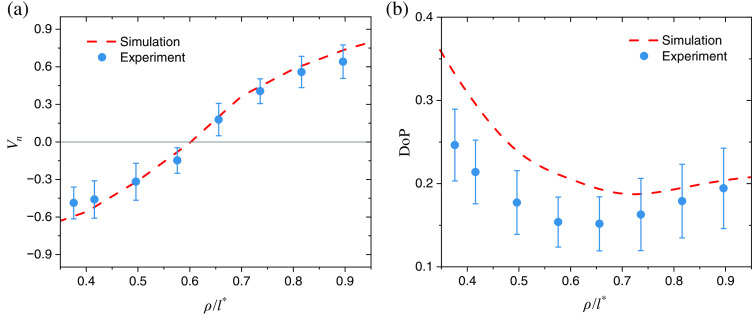
Comparison of (a) the normalized Stokes vector component $V_{n}$ and (b) the DoP values between the MC simulations (NA=30  deg, $\theta_{i}=55\;\deg$, $\theta_{d}=30\;\deg$) and the experimental measurements of tissue-mimicking phantom ($\mu_{s}=4\;{\mathrm{mm}}^{-1}$, $\mu_{a}=0.05\;{\mathrm{mm}}^{-1}$, g=0.8, n=1.46) performed with setup adopted from the previous works.^4^^–^^6^

All data present in Fig. 3 have been computed with open NA of the detector (NA>70  deg). In order to both explore the aperture influence and validate the results toward experimental data, another set of simulations was performed with aperture limited to NA=30  deg ensuring that only light photons meeting the condition $\mathrm{acos}(s_{N}\cdot s_{d})<\mathrm{NA}$ (see Sec. 3.1) are collected from the sample surface. From Fig. 4, we find good agreement of the MC simulations with experimental measurements performed with the setup described in previous works.^4^^–^^6^ Our simulation parameters provided in the beginning of the results section are already adjusted to approximately match the experimental setup configuration. In the experiment, we have carried out polarization measurements of RCP light scattered by thick phantom with known optical properties ($\mu_{s}=4\;{\mathrm{mm}}^{-1}$, $\mu_{a}=0.05\;{\mathrm{mm}}^{-1}$, g=0.8, n=1.46 at λ=640  nm)^62^.

We observe that limitation of the NA in the model led to the shift of the helicity flip location toward the source [$\rho /l^{*}∼0.6$ for NA=30  deg in Fig. 4(a) as opposed to $\rho /l^{*}∼0.8$ for open NA in Fig. 3(b)]. We also notice that, as shown in Figs. 3(b) and 3(c), vertical source–detector setup leads to the helicity flip position being shifted away from the source ($\rho /l^{*}∼1$), whereas angular source–detector placement causes helicity flip position to shift toward the source ($\rho /l^{*}∼0.8$). In other words, the larger $\theta_{i}$ and $\theta_{d}$ are, the closer helicity flip is to the source. Alternatively, this can be interpreted in terms of the medium refractive index n, which modifies the effective incident and detection angles $\theta_{i},\theta_{d}$ according to Snell refraction law. It should be also pointed out that depolarization composition of the backscattered CPL varies depending on the properties of turbid tissue-like disperse medium, such as its scattering characteristics, the size and composition of scattering particles implying different scattering phase functions, and the overall optical density^1^^,^^8^^,^^25^^,^^64^^,^^65^.

### 4.3 In-Depth Spatial Distribution of the CPL Components and Polarization Memory

Besides analysis of the surface response presented in the previous section, computer simulation provides an important insight on the in-depth light–tissue interaction. Sampling volume is a traditional parameter characterizing the detector depth sensitivity. Figures 3(a) and 3(f) show two-dimensional (2D) maps computed as difference between sampling volumes (SV) of the oppositely polarized RCP (blue) and LCP (red) light for several selected dimensionless source–detector separation distances $\rho /l^{*}$. With these maps, we demonstrate that $I_{R}$ and $I_{L}$ light portions statistically propagate at different depths within the sample, as suggested in previous works of Sridhar and da Silva.^66^ This result is well pronounced in the angular source–detector configuration [see Fig. 3(a)]. An important outcome is the possibility to tune the penetration depth of both left- and right-polarized components of light via adjusting angle and position of the source–detector configuration. It can be clearly seen that prior to the helicity flip point $I_{L}>I_{R}$ [Fig. 3(a) for $\rho /l^{*}=0.4$, Fig. 3(f) for $\rho /l^{*}=0.4,0.8$], and after the flip $I_{L}<I_{R}$ [Figs. 3(a) and 3(f) for $\rho /l^{*}=1.2$] in agreement with the results discussed in previous section. This proves the self-consistency of the proposed MC model and supports the capability of the model to operate with depolarized light through considering fully polarized orthogonal states. In this work, sampling volumes have been computed with^16^^,^^17^

$$\mathrm{SV}(r^{\prime })=\frac{\overset{N_{\mathrm{ph}}}{\underset{j=1}{\sum }}L_{j}(r^{\prime })I_{N_{j}}}{L_{0}\overset{N_{\mathrm{ph}}}{\underset{j=1}{\sum }}I_{N_{j}}}.$$ (34)

Here, $I_{N_{j}}$ corresponds to the detected intensity of the j’th-th MC-photon defined by the expression under the sum sign, i.e., in Eqs. (23) and (24), $N_{\mathrm{ph}}$ is the amount of detected photons, $L_{j}(r^{\prime })$ is a path length of the j’th-th MC-photon within a voxel centered at $r^{\prime }$, and $L_{0}$ is linear size of the voxel. Evaluation of Eq. (34) provides us with a 3D array SV(x,y,z) depicting detector depth sensitivity within each voxel. 2D maps shown in Figs. 2(a) and 2(f) are computed as ${\mathrm{SV}}_{R}(x,0,z)-{\mathrm{SV}}_{L}(x,0,z)$ with ${\mathrm{SV}}_{R},{\mathrm{SV}}_{L}$ defined via corresponding $I_{R},I_{L}$ intensities. To the best of our knowledge, this is the first time when the discussed phenomena of right- and left-polarized light components possessing different sampling volumes is both quantitatively and qualitatively described with the MC simulations.

**Fig. 5:**
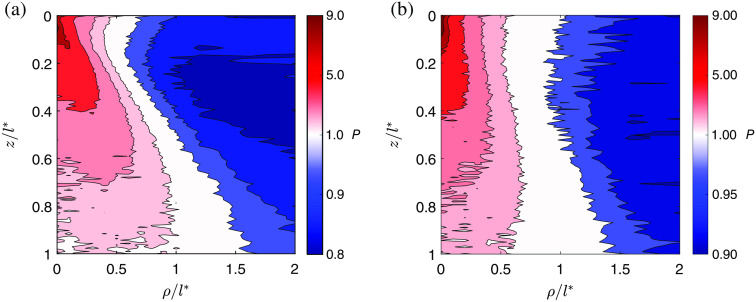
Polarization memory $P=I_{L}/I_{R}$ for (a) the angular setup with $\theta_{i}=55\;\deg$, $\theta_{d}=30\;\deg$ and for (b) the vertical setup with $\theta_{i}=\theta_{d}=0\;\deg$ as a function of the dimensionless penetration depth $z/l^{*}$ and source–detector separation $\rho /l^{*}$, where $l^{*}$ is the transport length. Scale step on the colorbar for regions with preserved helicity (blue) is chosen differently from the scale for regions with flipped helicity (red) in order to make the distribution details visible.

To conclude this section, we point out that within our model it is possible to extensively study the distribution of polarized light within tissue in terms of polarization extinction ratio (PER):^67^ $P=I_{L}/I_{R}$. PER characterizes the extent of polarization cross talk between flipped and preserved components of the backscattered CPL. Figure 5 shows the in-depth spatial distribution of the polarization memory, presented by analogy to the photon-measurement density function,^68^ in terms of gradient of PER computed similarly to the sampling volume in Eq. (34). PER refers to the relative intensities of LCP and RCP components and describes the mixing of flipped polarization with the orthogonal one as a result of multiple scattering interactions. Figure 5 shows a strong localization of LCP component in relation to the incident polarization state at the short ($\rho <l^{*}$) source–detector distances for both setup configurations. The linear polarization, emerged as a superposition of LCP and RCP components, demarcates areas of their localization. The wide aperture of the detector (NA>70  deg) and anisotropy of scattering g result in a broad range of scattering angles of photons and their path length distribution, leading to an asymmetry of the in-depth spatial distribution, which is strengthened when both source and detector are not oriented along the normal to the surface of the turbid medium.

### 4.4 Mueller Matrix Evaluation

**Fig. 6:**
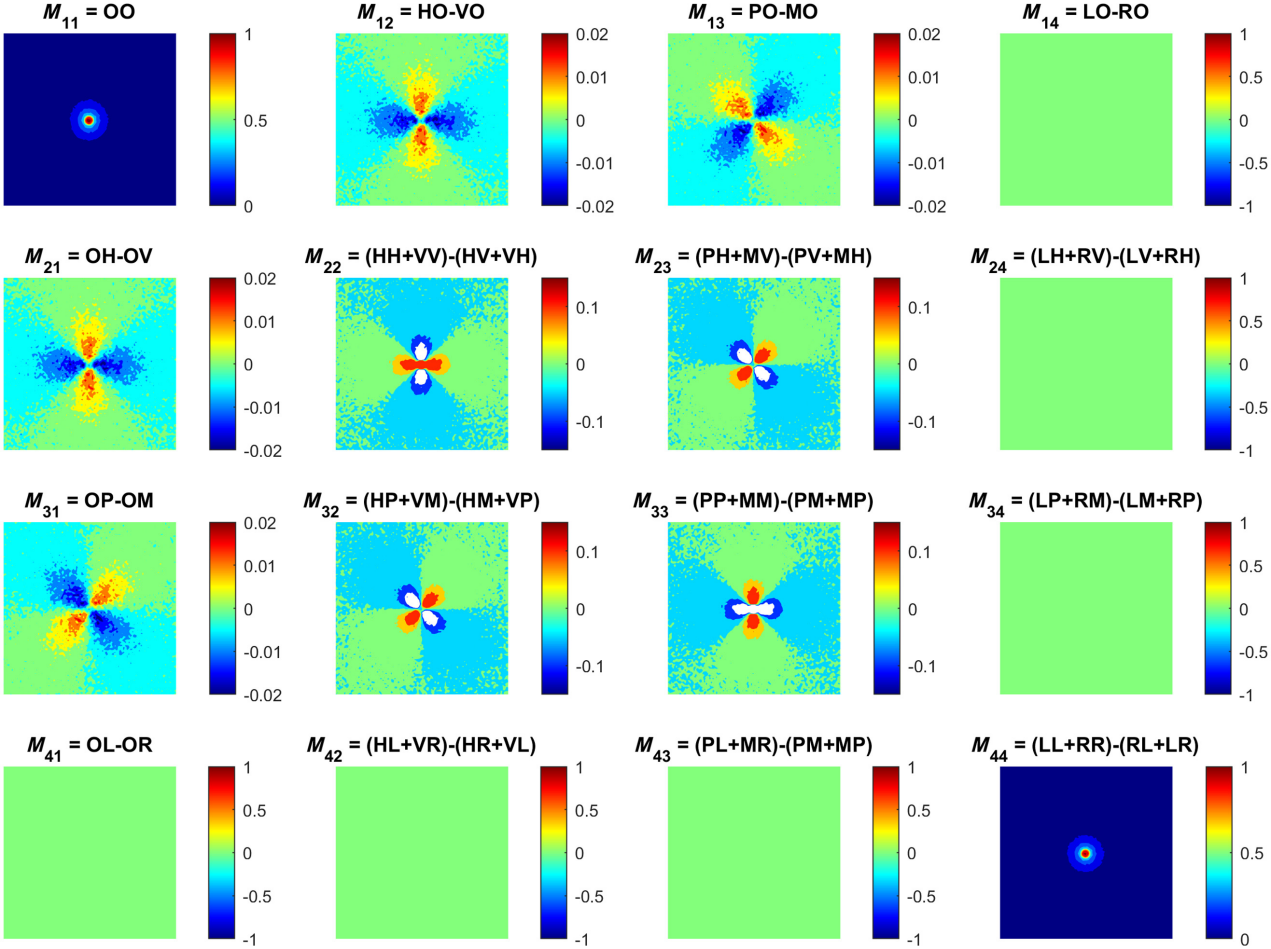
Mueller matrix elements obtained by MC modeling for turbid scattering medium with the following optical properties: $\mu_{s}=1\;{\mathrm{mm}}^{-1}$, $\mu_{a}=0.01\;{\mathrm{mm}}^{-1}$, g=0.74, n=1.33. Here, the detector registers the signal transmitted through medium with 4 mm thickness. The dimension of each image is 1×1  cm, which is equal to the detector size. The individual images are represented by a two-letter combination that denotes the input polarization and the output analyzer orientation as defined in Eq. (31).

Finally, in Fig. 6, we present an example of Mueller matrix elements computed by Eqs. (32) and (33). This data were obtained for the vertically positioned source and detector. Here, the detector registers the transmitted signal in 1×1  cm area, ρ=0. These results demonstrate that our developed approach is inherently capable of carrying out Mueller matrix computations. The ability to simulate Mueller matrix numerically is especially relevant because most of the experimental research on interaction of the polarized light with tissues employs Stokes–Mueller formalism as a standard.^61^^,^^69^ As outlined in Sec. 3.4, one of the main advantages of our approach is the ability to evaluate Mueller matrix without the need to launch multiple simulations for different incident polarization states. By presenting the established model in this paper, we aim to further develop our Mueller matrix MC with respect to applications in the course of the subsequent research.

## 5 Conclusion

We introduce an MC modeling approach that provides combined Jones and Stokes–Mueller formalism. Our model utilizes the polarization tracing framework based on the iterative solution to Bethe–Salpeter equation. The reflection and refraction of the linearly, elliptical, and/or CPL at the medium surface are generalized and properly included in the model. Self-consistency of the proposed model is ensured by the developed theoretical framework and confirmed by both numerical experiments and phantom measurements. One of the main advantages of the proposed approach is the ability to evaluate Mueller matrix elements, as well as other characteristics, such as sampling volumes or degrees of polarization, with single simulation.

The results of modeling studies reveal the origins of the experimentally observed helicity flip that depends both on the configuration of the source–detector setup and turbid medium properties. First, we have shown that for the CPL backscattered from the turbid medium the flipped helicity survival is prevailed at the short source–detector separation ($\rho <l^{*}$). A transition from LCP to RCP is revealed for longer distances ($\rho >l^{*}$) resulting in preservation of the helicity of incident light. Second, we have demonstrated that backscattered CPL within MC is appropriately decomposed into two fully polarized orthogonal components with opposite helicities, and their polarization state is fully defined. Third, we have reported on the different penetration depth of RCP and LCP light as demonstrated by the sampling volume simulations. And finally, we have addressed the in-depth binding of circular polarization memory with the helicity flips occurring within the medium.

It should be pointed out that developed MC framework is suitable to imitate light beams of different shapes, such as traditional point sources, plane waves, Gaussian and Bessel beams, as well as complex laser beams carrying OAM (e.g., Laguerre–Gaussian) via appropriate definition of the initial MC-photon intensity and direction distributions. In addition, diverse source–detector configurations, coherent properties of incident light and arbitrary polarization states can be taken into account without further modifications of the code core components.

In summary, the combined use of Jones and Stokes–Mueller formalisms in MC modeling offers benefits, such as comprehensive polarization modeling, flexibility in simulating different optical elements, accurate representation of complex optical systems, validation against experimental data, and enhanced understanding of polarization phenomena. These advantages make this approach valuable in a wide range of fields, including biomedical optics, remote sensing, atmospheric optics, and more.

## Data Availability

Data underlying the results are available from the corresponding author upon reasonable request.
